# Insights into platelet factor 4-derived peptide macrocycles; the mechanistic basis of their rapid and selective antiplasmodial actions

**DOI:** 10.1007/s00018-025-05757-y

**Published:** 2025-06-09

**Authors:** Dianne W. Xu, Karoline Raven, Sarah R. Woodcock, Bruce Munro, Isabella R. Palombi, Caitlin L. Gare, Andrew M. White, Lara R. Malins, Nicole Lawrence, Brendan J. McMorran

**Affiliations:** 1 Division of Immunology and Infectious Disease, The John Curtin School of Medical Research, Australian National University, Acton, Canberra, 2601 Australia; 2 Research School of Chemistry, Australian National University, Acton, Canberra, 2601 Australia; 3 Australian Research Council Centre of Excellence for Innovations in Peptide and Protein Science, The Australian National University, Canberra, ACT 2601 Australia; 4 Institute for Molecular Bioscience, Centre of Excellence for Innovations in Peptide and Protein Science, The University of Queensland, St Lucia, Queensland 4067 Australia; 5 Present Address: Institute for Molecular Bioscience, The University of Queensland, St Lucia, Queensland 4067 Australia

**Keywords:** Host defense peptide, Membrane-active peptide, Antiplasmodial peptide, Cell internalization, Microbe-host cell selectivity

## Abstract

The malarial parasite *Plasmodium* can acquire resistance to most mainstay antimalarial drugs, necessitating the development of new antiplasmodial agents with different modes of action. The innate defense protein, human platelet factor 4 (PF4), has a unique antiplasmodial action that involves selective entry into *Plasmodium*-infected red blood cells (RBC) and subsequent destruction of the parasite’s digestive vacuole (DV). This activity is recapitulated in PF4-derived internalization peptides (PDIPs). Here, we characterized the actions of PDIP analogs and PF4 in live *P. falciparum*-infected human RBC to understand their kinetics, effects on cell and parasite viability, and molecular requirements for antiplasmodial activity. The entry and accumulation of PDIP, and peptide-induced DV destruction, were distinguishable as ordered and rapidly occurring events that were equivalent to PF4. Both host cell and parasite plasma membranes remained intact and undamaged following destruction of the DV, although modest changes in phosphatidylserine (PS) exposure on the surface of the host cells (indicative of changes to its phospholipid organization) and swelling (but not lysis) of the intracellular parasite were observed. PDIP retained its macrocyclic structure, and its activity depended on elevated levels of PS on the surface of infected versus uninfected cells. Neither the intramolecular disulfide bond of PDIP, nor the parasite’s nutrient and ion transporter functions were required. These actions on the parasite DV were not detected for other antiplasmodial drugs and compounds. In conclusion, this study reveals the unique, rapid, and distinct antiplasmodial actions of PDIP, highlighting its potential for future antimalarial development.

**Supplementary Information:**

The online version contains supplementary material available at 10.1007/s00018-025-05757-y.

## Introduction

Malaria is an infectious disease caused by the protozoan parasite *Plasmodium* that is endemic to many countries in tropical regions. In 2022, an estimated 249 million cases and 608,000 malaria-related deaths occurred across 85 countries [1]. Despite significant advancements in malaria control over the past two decades, the continued emergence of drug-resistant parasite strains critically threatens eradication efforts [2, 3]. Particularly concerning are the new *P. falciparum* mutations that confer resistance to frontline artemisinin-combination therapies (ACTs) [4, 5], which have spread widely in Southeast Asia [6, 7] and recently in parts of Eastern Africa [8–10]. As such, there is an urgent need for new antimalarials that leverage novel mechanisms to target and kill malarial parasites.

Antimicrobial peptides (AMPs) are produced by a wide range of organisms to defend against invading pathogens [11]. Many, including human derived compounds, are gaining attention as anti-infective therapeutics for their unique mechanisms of action and potential to circumvent traditional drug resistance pathways [12–14]. Amongst several classes of AMPs, those that act on microbial targets by binding to and damaging the plasma membrane architecture, or traversing membranes and disrupting intracellular processes [15–17], are especially attractive for therapeutic development. Such AMPs exhibit a strong binding preference for membranes with a negatively charged outer surface, a universal feature of microbial prokaryotes, but do not bind to neutral membrane surfaces that are typical of healthy mammalian cells, thus enabling cell-selective targeting [12]. This selective binding and activity against negatively charged membranes is achieved by the characteristic amphipathic arrangement of charged and hydrophobic amino acids within AMP sequences.

Human platelet factor 4 (PF4) is a chemokine molecule produced primarily by platelets that participates in a range of hemostatic and immunomodulatory functions [18, 19]. It belongs to a multi-species protein family called kinocidins, characterized by the presence of chemokine domains that interact with their cognate receptors to elicit various immunological activities, and amphipathic AMP-like domains that are responsible for cytotoxic activity against a range of bacterial, fungal and protozoan organisms [20, 21]. In malaria infection, PF4 is the key mediator in the platelet-directed killing of asexual blood stage *Plasmodium*. Upon exposure to parasitized red blood cells, PF4 is released from platelet intracellular granules, is internalized via a Duffy antigen receptor for chemokines (DARC)-dependent process, and accumulates within the parasite cytosol where it destroys the digestive vacuole (DV), leading to parasite death [22–24]. These antiplasmodial properties of PF4 are attributed to short (14 amino acid) AMP-like segments that assemble into two head-to-tail amphipathic helices in the native PF4 tetramer [23, 25].

In previous work we developed a macrocyclic helix-loop-helix peptide, called cyclic PF4 peptide dimer (cPF4PD), composed of two PF4 AMP-like helices joined at one end by a flexible linker and at the other by a disulfide bond, which enters and kills *P. falciparum* with low micromolar potency [26]. cPF4PD and subsequent rationally designed analogs [27], herein collectively called PF4-derived internalization peptides (PDIPs), bind to and lyse synthetic membranes that are rich in negatively charged phospholipid headgroups. This lipid-binding specificity corresponds to the characteristic composition of *P. falciparum*-infected RBC membranes, which have more negatively charged phosphatidylserine (PS) exposed on the cell surface than healthy uninfected cells [28–30], as well as the parasite’s DV membrane, which is rich in negatively charged phosphatidylinositol 3-phosphate [31], thereby possibly explaining the antiplasmodial actions of PDIP. However, studies of other membrane-active AMPs suggest that additional factors could affect PDIP selectivity and cytotoxicity [13], including parasite-expressed nutrient and ion transporter channels that may mediate PDIP uptake [32, 33], and the reducing environment of the intraerythrocytic parasite cytosol [34, 35], which may affect the intramolecular disulfide bond and therefore, the peptide cyclization state.

Here, we expand the molecular understanding of the antiplasmodial activity associated with two near identical representative PDIP analogs, PDIP-1 and PDIP-3 (compounds **1** and **3** from ref [27], see Figure S1), and PF4. In live cells we observed rapid entry of an A488 fluorophore-labeled PDIP-3 derivative (PDIP-A488) into *P. falciparum*-infected but not uninfected RBCs, and then almost immediately after internalization, destruction of the parasite DV and swelling (but not lysis) of the parasite (using PDIP-1, PDIP-3 or PF4). PDIP-3 also increased the PS levels in the plasma membrane surface of infected host cells, indicative of changes to its phospholipid organization. Targeted entry of PDIP-A488 was dependent on levels of PS in the exposed outer leaflet of infected host cell membranes (elevated compared to uninfected cells), but not on the parasite nutrient and ion transport systems examined herein. Unlike PF4, PDIP-1 did not require DARC for its uptake and DV-destructive antiplasmodial activity. In contrast, none of more than 100 other antiplasmodial compounds and drugs examined disrupted the parasite DV. Further studies using PDIP-3 also revealed that maintaining the critical PDIP macrocyclic structure but not the redox sensitive disulfide bond was important for uptake and cytotoxicity. Collectively this study demonstrates the PDIP molecular scaffold as a remarkably specific and rapid-acting class of antiplasmodial, which combined with its unique human host-defense protein origins and mode of action, indicates its potential for further development into antimalarial drugs.

## Methods

### Peptide synthesis

PDIP analogs 1 and 3 (PDIP-1 and PDIP-3) were synthesized using automated Fmoc solid phase peptide synthesis, then purified using reverse phase HPLC as described in previous studies [26, 27]. PDIP-24 was synthesized as a linear hydrazide with an N-terminal Cys, then cyclized using native chemical ligation and desulfurized to convert the Cys to Ala, as previously described [27]. To produce PDIP-A488, a variation of PDIP-3 with an azidoalanine replacing serine in the flexible linker region was synthesized and purified as above. The A488 flurophore was incorporated via copper catalyzed azide alkyne cycloaddition of the peptide azide with alkyne-A488 [36]. The peptide sequences are shown in Figure S1.

### Parasite culturing

*P*. *falciparum* asexual blood stage parasites were cultured in O^+^ human RBCs (2.5% haematocrit) and complete culture medium (CCM), comprised of RPMI 1640 supplemented with 8.8 mM D-glucose, 22 mM HEPES, 208 nM hypoxanthine, 46.1 nM gentamicin, 2.8 mM L-glutamine (all from Sigma-Aldrich, Castle Hill, Australia), 2.1 g/L AlbuMAX® I (ThermoFisher Scientific, Australia) and 4.2% (v/v) O^+^ human serum. In some experiments the CCM contained TES (N-tris(hydroxymethyl)methyl-2-ammonioethanesulfonate) in place of HEPES at the same concentration (CCM-TES). The RBCs and serum were provided by Australian Red Cross Lifeblood, obtained from anonymous blood donors (aged 18–60 years). Cultures were maintained in sealed culture flasks with a gas mixture (1% O_2_/5% CO_2_/94% N_2_) and kept in an orbital shaking incubator at 50 rpm at 37 °C. Culture parasitemia was maintained between 0.2% and 10% and CCM was changed every 1–3 days. Trophozoite stage parasites used in mass spectrometry and live parasite imaging studies were generated by magnet enrichment (see below). Whereas for the parasite growth and flow cytometry peptide uptake assays, trophozoite stage parasites were obtained by treating culture cell pellets (isolated by centrifugation 500 *g* for 5 min at room temperature) with sorbitol (10 × cell pellet volume 5% w/v D-sorbitol for 10 min at room temperature, followed by washing and replenishing with new CCM), performed 16–20 h prior to the experiment.

### Live parasite imaging studies

Cultures containing synchronized trophozoite stage parasites (*P. falciparum* expressing PMII-GFP) were isolated by centrifugation and resuspended in serum-free CCM (SF-CCM, 10 × cell pellet volume). A varioMACS CS column (Miltenyi Biotec) was equilibrated with SF-CCM (2 × column volume), placed next to a strong magnetic field and the isolated parasites applied, followed by chasing with SF-CCM (approximately 2 × column volumes) until the outflow of RBCs had ceased. The column was then removed from the magnet and additional SF-CCM applied (approximately 1 × column volume) to elute the previously retained trophozoites, which were collected by centrifugation and kept in SF-CCM (5 × eluted cell pellet volume) at 37 ˚C until the imaging studies (performed within 1 h of the column purification). These preparations typically contained between 50–70% infected RBCs (mainly late stage trophozoites and schizonts, > 30 h post cell invasion) and uninfected RBCs (30–50%). To image the infected cells, glass slides were coated in 0.1% polyethyleneimine (to aid cell adherence) and a hydrophobic pen was used to draw an approximately 1 cm^2^ area circle (to contain the sample). Aliquots of cells (5–10 µL) were mixed with treatment compound (or left untreated as controls) and a 2 µL volume immediately added to the slide, overlaid with a glass coverslip (22 mm^2^) and viewed using an Axio Observer inverted microscope (ZEISS) at 630 × magnification. A field of view (typically containing 10–20 infected and uninfected cells) was identified, and images of the same field captured every 3 s over a time course, ranging between 10 min (DV destruction studies) to 30 min (parasite swelling studies), using differential interference contrast (DIC) and GFP channels. Videos of the image frames were generated and analyzed using ZEN Software (ZEISS). Criteria for including cells in the analysis were: visible and separately defined RBC and parasite membranes, and PMII-GFP fluorescence either localized to a distinct area of the cytoplasm and surrounding a hemozoin crystal (an intact DV) or filling the parasite cytosol (a destroyed DV). Active DV destruction events were identified by changes in the localization of GFP fluorescence from within an intact DV to filling the parasite cytosol. Parasites in which GFP was localized to the cytosol when the imaging commenced were deemed to have already undergone DV destruction before recording began. The numbers of parasites with a destroyed DV were quantified in 1 min intervals from the start of recording. Data were grouped according to different observation fields (each involving an independent treatment and/or independent parasite preparation) and the proportions of intact and destroyed DVs at each minute in each field. Cell areas were determined in images collected from the DIC channel using ZEN software area object tools.

### Peptide uptake and annexin V binding studies

Synchronized trophozoite stage parasites (*P. falciparum* strain 3D7, 3–5% parasitemia) were isolated by centrifugation, resuspended at 2% hematocrit in phosphate buffered saline (PBS, 137 mM NaCl, 2.7 mM KCl, 10 mM Na_2_HPO_4_, 1.8 mM KH_2_PO_4_) containing 1% bovine serum albumin (both from Sigma Aldrich, PBS/BSA) and 5 µg/mL Hoechst 33342 (Thermo Fisher Scientific), and incubated in the dark at 37 °C for 15 min. Aliquots were then mixed with prepared dilutions of PDIP-A488 and immediately subjected to flow cytometry at multiple time points (between 1 and 60 min). An LSR Fortessa cell analyzer (BD Biosciences) was used to analyze approximately 100,000 cells per sample and FlowJo software to identify infected and uninfected cells (Hoechst 33342^+^ where parasite DNA is present), peptide containing cells (A488^+^) and relative peptide levels in each cell type (A488 mean fluorescence intensity, MFI). Uninfected, unstained and untreated cells were used to define negative populations (Figure S3). In some experiments, cells were pretreated with compounds (vanadate, A23187, annexin V, furosemide, 5-nitro-2-(3-phenylpropylamino) benzoic acid (NPPB), concanamycin A or cipargamin, all from Sigma Aldrich, see main text and figures legends for specific conditions) and extensively washed (three times in 1% BSA/PBS using 10 × cell pellet volumes) prior to Hoechst staining and peptide treatment. Annexin V binding studies were performed by resuspending parasites in BSA/PBS containing 2 mM CaCl_2_, followed by addition of 5% (v/v) FITC Annexin- V conjugate (BD Bioscience) and incubation for 30 min in the dark at 37 °C, staining with Hoechst 33342 and flow cytometry analysis.

### Peptide mass spectrometry studies

Trophozoite stage parasites (*P. falciparum* strain 3D7, cultured in CCM-TES) were enriched by magnetic purification and adjusted to 10% hematocrit in serum-free CCM-TES. Aliquots (10 µL containing approximately 1 × 10^7^ cells) were incubated with PDIP-3 (10 µM for 5 min at 37 °C) and then washed twice in 1 mL ice-cold PBS. The cell pellets were lyzed in 30 µL distilled water, and then immediately mixed with 60 µL HPLC-grade acetonitrile/2% trifluoroacetic acid (TFA) and incubated for 10 min at 4 °C to precipitate cell and parasite proteins and solubilize small molecules and peptides. The soluble fraction obtained after centrifugation (21,000 *g* for 5 min at 4 °C) was subjected to iodoacetamide alkylation to prevent re-oxidation of any reduced PDIP-3 (addition of 70 µL of a 100 µM solution of iodoacetamide in acetonitrile and incubated for 30 min at 4 °C) followed by TFA treatment (addition of 20 µL of 6% TFA in acetonitrile, 10 min at 4 °C) and centrifugation. Aliquots of the recovered supernatant (55 µL) were added to glass vials with 145 µL 0.1% formic acid in MilliQ water for HPLC–MS analysis. A reduced PDIP-3 standard was prepared by reduction (in tris(2-carboxyethyl)phosphine), followed by alkylation and recovery as described above. The oxidized (disulfide cyclic) PDIP-3 standard consisted of unmodified peptide suspended in 1% formic acid.

Chromatographic separation of peptides was achieved on an Agilent ZORBAX C3 column (4.6 × 50 mm, 3.5 µm particle size) attached to the loading pump of an ultra-high performance liquid chromatograph (UHPLC) (Dionex Ultimate 3000 RSLC Nano). It consisted of a two-component system with mobile phase A (0.1% formic acid in MilliQ water) and mobile phase B (0.1% formic acid in acetonitrile) at a flow rate of 0.5 mL/min. The HPLC gradient was: 5% B to 15% B over 0.25 min, followed by a 6.50 min wash at 80% B and a 5 min re-equilibration at 5% B. The column was maintained at 40 °C and the injection volume was 5 µL. The diverter valve was used for the first 4 min to divert any excess CCM or buffers from the mobile phase to waste. The sampler needle was washed in 500 µM 0.1% formic acid/MilliQ water (50 µL at 4 µL/s) after each sample to ensure no carryover. Acquisition was executed in positive electrospray ionization mode with the full MS scan acquired in the Orbitrap Fusion ETD instrument (Thermo Fisher Scientific) in data-dependent acquisition (DDA) mode using the orbitrap mass analyzer for MS1 and MS2 with a resolution of 50,000 FWHM for MS1 and a *m/z* scan range 200–2000. MS1 microscans were set to 10 with a cycle time of 1 s. The heated electrospray ion source had a static spray voltage of 3500 V. Sheath gas, aux gas and sweep gas were set to 50, 10 and 1, respectively (arbitrary units). The vaporizer temperature and ion transfer tube temperatures were 350 °C and 325 °C, respectively. Samples with peak intensities higher than 5.0 × 10^4^ were selected for fragmentation and fragmented ions were analyzed in the orbitrap at a resolution of 15,000 using HCD fragmentation with fixed collision energy of 30% and using 2 microscans. To determine the exact retention time of the iodoacetamide alkylated and cyclic peptide extracts, 1 pmol standards of each peptide were used as controls and subjected to the same HPLC–MS method. Peptide proton adducts with charge states 3^+^, 4^+^, 5^+^ and 6^+^ ([M + 3H]^3+^*,* [M + 4H]^4+^, [M + 5H]^5+^, [M + 6H]^6+^) were assigned using exact mass (< 5 ppm) and retention time. Manual raw data evaluation and initial identification of peptide signals was performed using Freestyle software (Thermo Fisher Scientific, version 1.6.75.20). Automated integration of target peptide peak areas was performed using the quantitative processing tools in the Xcalibur software package (Thermo Scientific, version 4.3.73.11). Peak integration was performed on the peptide MS1 signals using the Genesis algorithm with a retention time window of 30 s.

### Parasite growth inhibition assays

Trophozoite stage parasites (*P. falciparum* strain 3D7) were isolated by centrifugation and resuspended in uninfected blood and SF-CCM to produce a 0.5% parasitemia and 2% hematocrit. Serial dilutions of test peptides were prepared in SF-CCM (5 × final concentration) and then mixed with parasites at 1:5 dilution ratio (10 µL + 40 µL) in 96 well round bottom plates, and incubated in an airtight container filled with culture gas mix at 37 °C for 48 h. The cells were then fixed in 1% (w/v) formaldehyde (3:1 v/v PBS:Cytofix™, BD Biosciences) for at least 24 h at 4 °C, washed in PBS, stained with 5 µg/mL Hoechst 33,258 in PBS/BSA for at least 15 min at 4 °C. Fluorescence signals of stained cells were measured using a LSR Fortessa cell analyzer (BD Biosciences) with at least 200,000 events (cells) collected per sample. Percentages of infected cells were identified and computed using FlowJo software (BD Biosciences). The IC_50_ data was determined for each treatment with Prism (GraphPad Software) using nonlinear regression of a dose–response curve.

## Results

### PDIP internalization and accumulation kinetics

**Fig. 1:**
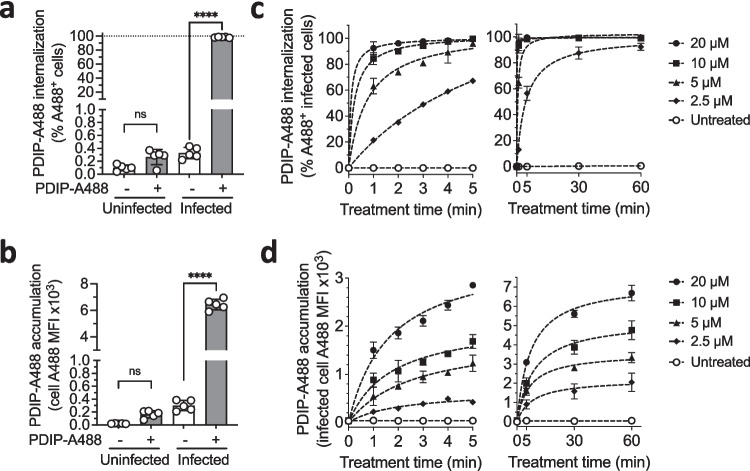
Internalization and accumulation of PDIP-A488 in *P. falciparum* infected and uninfected RBC. **a**. and **b**. PDIP-A488 internalization (% A488-positive cells, A488^+^) and accumulation (cell A488 mean fluorescence intensity, MFI) in *P. falciparum* 3D7 infected and uninfected red blood cells after 60 min incubation with or without 20 µM PDIP-A488. Shown are the measures from five independent experiments, means (histogram bars) and SD (error bars). Comparisons of peptide-treated versus untreated cells were determined using one-way ANOVA with Holm-Sidak’s correction for multiple comparisons; ns, *p* > 0.05; **** *p* < 0.0001. **c**. and **d**. PDIP-A488 internalization and accumulation in infected cells after 1–5 min and 5–60 min treatment with different concentrations PDIP-A488 or no peptide (untreated). Shown are the means from three independent experiments (dot symbols) and SD (some error bars are obscured by data points). Fitted curves (dashed lines) were calculated using nonlinear regression

Previous studies have demonstrated that PF4-derived peptides (and PF4) enter and accumulate in intraerythrocytic parasites and that uninfected cells are resistant to peptide internalization [23, 26]. Here we developed a protocol to measure peptide internalization into live cells in real time, enabling us to examine uptake kinetics of a prototypical PDIP, PDIP-3 (Figure S1), and factors that may regulate the process. In vitro cultures comprised of *P. falciparum* strain 3D7-infected (synchronized trophozoite stage) and uninfected RBCs were incubated with an Alexa Fluor 488-labelled PDIP-3 derivative (PDIP-A488) (Figure S1), which had comparable antiplasmodial activity to free PDIP-3 (Figure S2). Treated cells were analyzed using flow cytometry to identify cells with internalized PDIP-A488 (A488^+^), and the A488 mean fluorescence intensity (MFI) was used to report on total levels of PDIP-A488 accumulation in each cell type (Figure S3). In infected RBCs, 60 min treatment with 20 µM PDIP-A488 resulted in 100% A488^+^ cells, and the A488 MFI was 30-times higher (compared to untreated cells, which represented the background MFI). In contrast, no internalization or accumulation of PDIP-A488 were detected in uninfected RBCs (Fig. 1a and b). Thus, PDIP-A488 was selective for infected cells.

In further experiments a range of PDIP-A488 concentrations (2.5–20 µM) and incubation times (1–5, 30 and 60 min) were used to study the internalization and accumulation kinetics in infected cells. Internalization of PDIP-A488 occurred rapidly with all the treatment concentrations, although the rate of uptake was noticeably less with the lowest concentration (2.5 µM) compared to the three higher concentrations (5, 10 and 20 µM) (Fig. 1c). Using linear regression analysis, the estimated times to reach 100% A488^+^ infected cells was between 5–7 min when 5, 10 and 20 µM PDIP-A488 were added, and 40 min for 2.5 µM PDIP-A488 (Table S1). Accumulation of PDIP-A488, in contrast, was detectable during the first few minutes of treatment, but continued to occur for longer times, at least up until 60 min (the longest incubation time examined) (Fig. 1d). While the predicted maximal accumulation levels (maximal A488 MFI, determined by linear regression) were relative to the treatment concentration, they were not directly proportional. For example, the difference in the maximal A488 MFI between the 5 µM and 20 µM treatments (a four-fold concentration difference) was only two-fold (Table S1). Collectively, these observations suggest that PDIP-A488 internalization and accumulation both occur rapidly but are distinct processes with different kinetics. In addition, the treatment concentration versus maximal accumulation findings suggest the peptide enters infected cells against a concentration gradient. By deduction, it is possible that either active processes in the cell drive peptide entry, or that initial exposure to PDIP may in some way enhance the cell’s susceptibility to peptide uptake, both of which we investigated below.

### Characterization of factors required for PDIP internalization

#### PS exposure on *P. falciparum*-infected RBCs

We hypothesized that PDIP analogs selectively enter infected RBCs because of cellular components that are specific or enriched in these cells. One possible component is the distribution of PS phospholipid in RBC membranes. We previously demonstrated that PDIP-1 (cPF4PD) interacts strongly with lipid bilayers that are rich in negatively charged phospholipid headgroups, including PS [26], and infection by *P. falciparum* is known to increase the levels of PS on the outer surface of infected RBCs [29, 30]. To further interrogate the possible relationship between PDIP selectivity for infected RBCs and levels of exposed PS, we first examined the levels of exposed PS on live *P. falciparum*-infected RBCs. Infected cells bound significantly more annexin V-FITC (a specific probe for membrane PS) than uninfected cells, which could be blocked by adding excess unlabeled annexin V (Figure S4a). Next, we generated both infected and uninfected RBCs with elevated membrane PS levels, by treating with 500 µM sodium orthovanadate (vanadate) or 2 µM A23187. Vanadate treatment modestly increased annexin V-FITC binding in both infected and uninfected cells (approximately 50% higher than untreated cells), whereas A23187 treatment caused a more substantial increase, with annexin V-FITC binding elevated eight times in infected RBCs and fourteen times in uninfected RBCs. Notably, we observed A23187 treatment resulted in levels of annexin V-FITC binding that were six times higher for treated uninfected cells compared to untreated infected cells (Figure S4b). Thus, in both infected and uninfected cells A23187 induced a greater extent of PS externalization on RBC membranes than vanadate. A23187 also enriched PS to greater degree than the parasite infection.

**Fig. 2:**
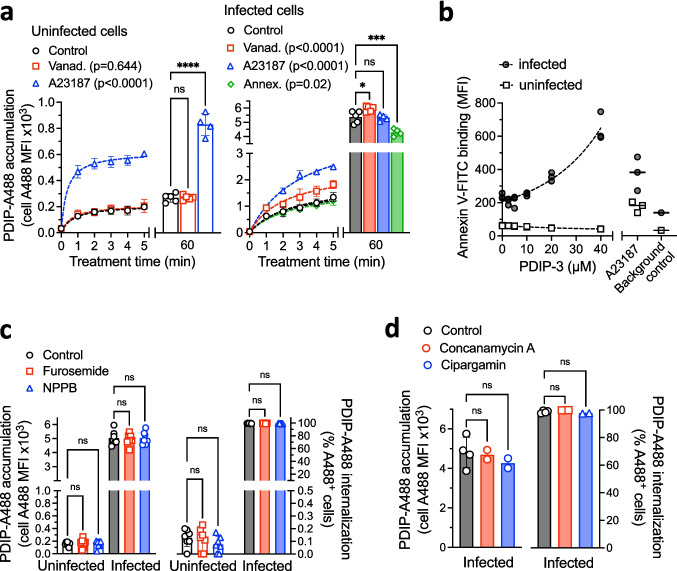
Factors determining PDIP-A488 peptide accumulation in *P. falciparum* infected RBC and effects of PDIP on phosphatidylserine levels in membranes of infected and uninfected RBC. **a**. PDIP-A488 accumulation (cell A488 mean fluorescence intensity, MFI) in uninfected and *P. falciparum* 3D7 infected red blood cells either untreated (control) or pretreated with 500 µM sodium orthovanadate (vanadate), 2 µM A23187, or 40 µg/mL annexin V (Annex., infected cells only) and washed before treating with 10 µM PDIP-A488 for between 1–5 min or 60 min. Shown in the left plots are the means from at least three independent experiments (dot symbols), SD (error bars) and fitted curves (dashed lines) calculated using nonlinear regression. In the right plots are measures from at least four independent experiments (dot symbols), means (histogram bars) and SD. Figure key includes p-values for comparisons between treatment versus control conditions during 1–5 min using a mixed model analysis. **b**. Annexin V-FITC binding levels (MFI) on infected and uninfected RBC after treatment (60 min) with 2.5–40 µM PDIP-3 or 2 µM A23187 determined in a representative experiment (showing treatment replicates and fitted curves using non-linear regression) and the MFI measured in absence of annexin V-FITC (Background control). **c**. PDIP-A488 internalization and accumulation in uninfected and infected RBC pretreated with 100 µM furosemide or 100 µM 5-nitro-2-(3-phenylpropylamino) benzoic acid (NPPB) for 60 min and then incubated with 10 µM PDIP-A488 for 60 min. **d**. PDIP-A488 uptake and accumulation in infected RBC pretreated with 200 nM concanamycin A or 5 nM cipargamin for 60 min and then incubated with 10 µM PDIP-A488 for 60 min. For **c** and **d**, shown are the measures from at least two independent experiments, means and SD. Comparisons to control cells determined using one-way ANOVA with Holm-Sidak’s correction for multiple comparisons; ns, *p* > 0.05; *, p < 0.05; ** *p* < 0.01; *** *p* < 0.001; **** *p* < 0.0001

Next, RBCs (infected and uninfected) were pretreated with either vanadate or A23187 (or left untreated as controls) and then incubated with 10 µM PDIP-A488 for 1–5 min and 60 min. Peptide accumulation was quantified by A488 MFI, as described above. No changes in A488 MFI were observed for the uninfected cells treated with vanadate over the 1–5 min incubation periods or after 60 min (representing peptide uptake and maximal accumulation, respectively), whereas significantly greater A488 MFI was observed for the A23187-treated cells during the 1–5 min period and after 60 min (approximately three times higher than control) (Fig. 2a, left panel). Thus, the A23187 treatment allowed PDIP-488 to rapidly accumulate inside normally peptide-resistant RBCs, suggesting that levels of exposed PS may be an important determinant for PDIP internalization. Analysis of the infected cells revealed that although the A488 MFI increased rapidly and to maximal levels that greatly exceeded those in uninfected cells (including 6 times more than in the A23187-treated uninfected cells), the effects of the vanadate and A23187 were more modest. PDIP-A488 uptake was only minimally enhanced and maximal accumulation was only 10% higher than in control cells. Collectively, these studies reveal that increased PS-exposure following treatment with A23187 greatly enhanced peptide uptake into uninfected cells, but not infected cells. PDIP-A488 uptake was also measured for infected cells pretreated with excess unlabeled annexin V to bind and block externalized PS from binding to annexin V-FITC (Figure S4a**)**. Blocking PS reduced the maximal A488 MFI by only 20% (Fig. 2a, right panel). Thus, differences in the PS levels between infected and uninfected cells were only a partial determinant underpinning the cell selectivity of PDIP-A488.

The infected and uninfected RBC were also treated with different concentrations of PDIP-3 (unlabeled) for 60 min and analyzed for levels of annexin V-FITC binding. In the infected cells PDIP-3 treatment resulted in higher annexin V-FITC levels, suggesting increased levels of exposed PS. The effect was greatest with the highest PDIP-3 concentration (40 µM), where levels were approximately three times higher than untreated cells (Fig. 2b). In contrast, PDIP-3 did not increase annexin V-FITC binding on uninfected RBC at any of the concentrations tested. Thus, PDIP-3 treatment (for 60 min) increased the levels of PS in the outer plasma membrane leaflet of infected but not uninfected RBCs.

#### New permeability pathway (NPP) and ionic composition

We were interested in determining whether PDIP-A488 uptake into infected RBCs requires the parasite-expressed new permeation pathway (NPP), a host membrane-localized transporter system established and used by intraerythrocytic *Plasmodium* to access nutrients and exchange waste products with the external medium and essential for parasite growth [32, 37]. The composition and molecular size of PDIPs fall outside the usual range for the NPP preferred substrate types (< 300 Da-sized anions and small organic solutes [38, 39]), however, we expected that the NPP might be involved because it is expressed throughout blood stage parasite development and is absent in uninfected RBC. To test this hypothesis, we treated infected and uninfected RBCs with two NPP inhibitors; furosemide and 5-nitro-2-(3-phenylpropylamino) benzoic acid (NPPB) [39] at concentrations which block the uptake of L-sorbitol, a well-established NPP substrate (Figure S5). Cells (infected and uninfected, and with or without pretreatment with NPP inhibitors) were co-treated with PDIP-A488 (10 µM). Cell populations with internalized peptide (A488^+^) were identified and A488 MFI was determined, as described above. As expected, the internalization (A488^+^ cells) and accumulation (A488 MFI) of PDIP-A488 were much greater in infected than uninfected cells, indicative of cell-selective peptide entry. However, the NPP inhibitors (furosemide and NPPB) did not have any effect, indicating that the NPP is not required for uptake and accumulation of PDIP-A488 (Fig. 2c).

*Plasmodium* parasites have a distinct ionic composition compared to their host cells, including reduced cytosolic sodium, and they maintain a proton gradient between the cytosol and parasitophorous vacuole. These factors may promote peptide uptake or retainment due to the high net positive charge on PDIPs. To investigate this possibility, infected RBC were treated with two different parasite ion-channel inhibitors, cipargamin and concanamycin A, to determine whether altering ionic determinants affects peptide internalization and accumulation. Cipargamin targets the sodium transporter P-type cation ATPase (PfATP4), increasing parasite cytosolic sodium and pH [40], whereas concanamycin A targets a V-type H^+^-ATPase and disrupts the parasite’s pH regulation and membrane potential [41]. Treated cells were incubated with PDIP-A488 (10 µM) and the proportions of A488^+^ cells and A488 MFI levels were compared to the previous experiments. Neither inhibitor caused any changes (Fig. 2d), therefore, the action of these ion transporters is not required for uptake and accumulation of PDIP-A488.

#### Requirement for cyclized versus non-bonded PDIP in uptake and parasite killing

**Fig. 3:**
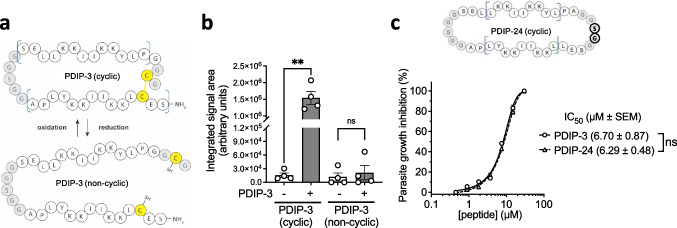
Internalized PDIP disulfide bond oxidation state and the anti-plasmodial activity of disulfideversus covalent-bonded PDIP. **a**. Cartoon representations of PDIP-3 in cyclic (oxidized) and non-cyclic (reduced) forms with its redox sensitive disulfide bond highlighted. Cys and disulfide bonds are shown in yellow, helical regions are indicated with blue brackets **b**. Relative levels of cyclic and non-cyclic PDIP-3 detected in *P. falciparum* 3D7 infected RBC after treatment with 10 µM PDIP-3 using HPLC–MS. See Figure S6 for details of HPLC–MS detection method. Shown are the measures from four experimental replicates, means (histogram bars) and SD (error bars). Comparisons determined using one-way ANOVA with Holm-Sidak’s correction for multiple comparisons; ns, *p* > 0.05; ** *p* < 0.01. **c**. Cartoon representation of amide-bonded analog PDIP-24 with the disulfide to amide bond substitution shown in bold, and *P. falciparum* 3D7 growth inhibition activities of PDIP-3 and PDIP-24. Shown are the means of three experimental replicates and fitted curves using non-linear regression. Shown in the figure key are the 50% inhibitory concentrations (IC_50_) and the comparison between the fitted curves determined using the extra sum of squares F test. ns, *p* > 0.05

The PDIP macrocyclic structure is stabilized by a disulfide bond between cysteine residues positioned at the N- and C-terminus. We previously reported that the disulfide bond was important for antiplasmodial potency, as a non-disulfide analog (PF4PD, non-cyclized and unstructured) was less active than its disulfide-stabilized cyclic counterpart (cPF4PD, PDIP-1) [26]. Recognizing the relatively strong reducing environment of the parasite cytosol [34, 35] and that the disulfide bond of macrocyclic PDIP analogs may be sensitive to reduction that would disrupt its structure (Fig. 3a), we investigated whether PDIP-3 undergoes reduction after internalization. A sensitive high performance liquid chromatography tandem mass spectrometry (HPLC–MS) method was developed to allow distinction between the disulfide-bonded (oxidized and cyclic) and non-bonded (reduced and non-cyclic) forms of PDIP-3 (Fig. 3 and Figure S6). Magnet-enriched (trophozoite stage) *P. falciparum* 3D7-infected RBC were treated with PDIP-3 (10 µM) or left untreated as a control, washed to eliminate non-internalized peptide, and then protein extracts were prepared and analyzed using HPLC–MS. The results showed a consistent presence of oxidized, disulfide-cyclic PDIP-3, whereas no reduced PDIP-3 was observed (Fig. 3b). We also investigated whether the peptide disulfide bond was necessary for parasite killing, considering the possibility that changes in its redox state (transient or otherwise undetectable in our HPLC–MS studies) may be necessary for its internalization or cytotoxic actions. To do this, we compared the parasite growth inhibitory activity of disulfide-bonded PDIP-3 with a backbone cyclic analog PDIP-24 (compound 24 from ref [27]), in which the disulfide bond was replaced by an amide bond to prevent loss of the cyclic structure in reducing environments (Figure S1, Fig. 3c cartoon). These disulfide-bonded and amide-bonded peptides performed similarly in parasite growth assays (Fig. 3c). Collectively, these findings suggest that disulfide-linked macrocyclic PDIP analogs retain their structure inside the parasite cytosol. The cyclic structure likely prevents reducing enzymes from parasite thioredoxin and glutathione systems [35] from accessing the disulfide bond. Moreover, the disulfide bond, or reduction to linearize the cyclic structure, are not required to kill the parasite.

### Real time imaging analysis of PDIP-induced destruction of the parasite DV

The DV-destructive actions of PF4 and PF4-derived peptides have been demonstrated previously using immunostaining and microscopy of fixed parasites [23, 26]. Here, we studied the phenomenon using real time fluorescence microscopy of live *P. falciparum* expressing GFP-tagged plasmepsin II (PMII-GFP), a soluble DV-localized protein. For these studies we used near-identical analogs PDIP-1 and PDIP-3 (see Figure S1). PDIP-1 lacks an N-terminal Gly residue but has equivalent parasite killing potency and low hemolytic activity, as well as comparable helicity, membrane binding affinity and resistance to breakdown by serum proteases, as compared to PDIP-3 [27]. Infected cells (magnet-enriched trophozoites) were treated with 20 µM PDIP-1 or PDIP-3, or left as untreated controls, and immediately examined using fluorescence microscopy. Time-lapse videos were prepared by capturing images of the same region of interest every 2–3 s for 10–30 min and used to document the temporal dynamics of DV loss, the morphology and integrity of host and parasite membranes, and the relative volume (area) of the parasites and cells.

**Fig. 4:**
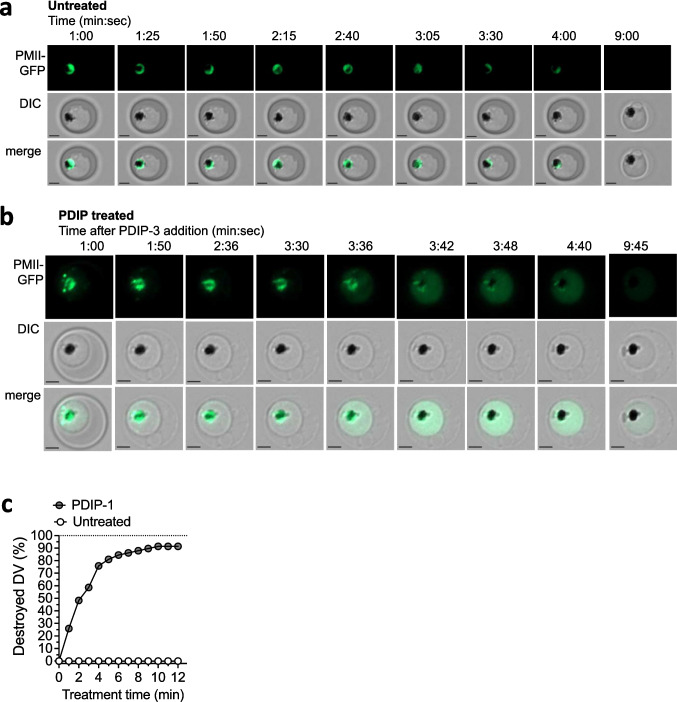
Video imaging analysis of PDIP-induced destruction of the parasite digestive vacuole. **a**. and **b**. Representative time lapse images of *P. falciparum* PMII-GFP infected RBC, either as untreated controls or after treatment with 20 µM PDIP-3, respectively. In each figure the same cell is shown at different incubation times viewed using GFP fluorescence (PMII-GFP) to visualize the digestive vacuole (DV) and differential interference contrast (DIC) to visualize the parasite and RBC. Note, the parasite in **a**. has shifted position and the RBC becomes slightly out of focus between 4 and 9 min. Scale bars indicate 2 µm. These images are derived from Videos S1 and S2, respectively. **c**. Proportions of parasites in infected RBC with a destroyed DV at different times after treatment with 20 µM PDIP-1, or untreated. For each respective condition, between 58 and 87 parasites were counted.

The untreated cells and parasites appeared viable and healthy under these conditions, as indicated by their visible integrity, including undamaged cellular membranes and an intact DV, which appeared as an intense GFP fluorescence surrounding the hemozoin crystal, albeit with gradual diminishment of the GFP signal likely due to photobleaching (Fig. 4a and Video S1). In contrast, treatment with PDIP-1 or PDIP-3 resulted in rapid dispersion of GFP fluorescence from the DV into the parasite cytosol within 2–10 min post-treatment (Fig. 4b and Videos S2-3). We interpreted this event as the permeabilization or destruction of the DV membrane and release of the organelle’s contents into the parasite cytosol. For cells treated with PDIP-1, destruction of the DV was evident in 81% (47/58) of parasitized cells within 5 min of treatment, and 91% (53/58) after 10 min (Fig. 4c). The GFP fluorescence remained localized within the parasite cytosol and did not spread into the host cell or surrounding medium, although decay of GFP fluorescence signal was observed in some cells, which was more rapid than in untreated control cells (compare Videos S2 and S1, respectively). It was presumed this loss of GFP fluorescence was a consequence of the parasite’s cytosolic and DV contents combining and increasing the photolabile or proteolytic conditions (e.g. altered cytosolic pH [26]).

**Fig. 5:**
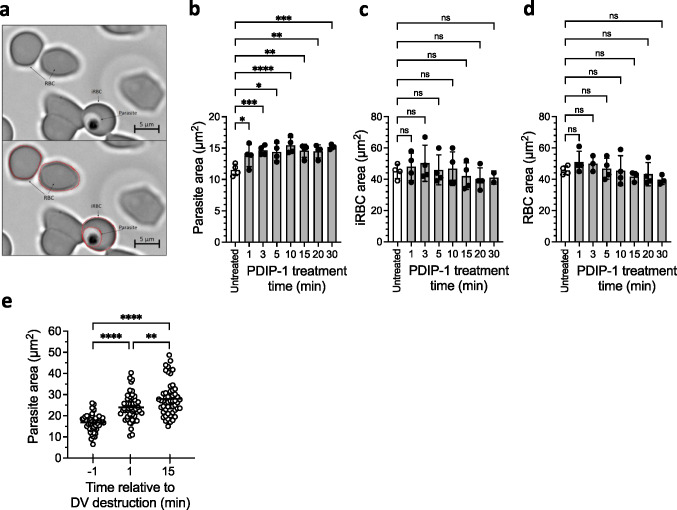
Analysis of the size of intracellular parasites, infected and uninfected RBCs following treatment with PDIP. **a**. Differential interference contrast (DIC) images showing an intracellular *P. falciparum* 3D7 (Parasite), an infected host RBC (iRBC) and uninfected RBCs (RBC). Each panel shows the same image. The lower panel includes outlines of the perimeters of the parasite, iRBC and RBCs (red circles), which were used to calculate the two-dimensional area of each. **b-d**. The respective areas in *P. falciparum* 3D7 parasites, iRBC and RBC compared before (untreated) versus different times after treatment with 38 µM PDIP-1. Shown are the measures from 4–5 independent experiments, means (histogram bars) and SD (error bars). **e**. Areas of *P. falciparum* PMII-GFP parasites treated with 20 µM PDIP-1, determined one minute before and one and fifteen minutes after DV destruction. Shown are the measures from 58 individual parasites and means (horizontal lines) from three independent experiments. For **b**-**e**, comparisons were determined using ANOVA with Sidak’s corrections for multiple testing. ns, *p* > 0.05; *, *p* < 0.05; **, *p* < 0.01; *** *p* < 0.001; ****, *p* < 0.0001

In cells undergoing DV destruction there was also noticeable increase in the size of the parasites (evident in Video S3). Separate studies were performed on samples of *P. falciparum* 3D7 (magnet-enriched trophozoites) in which the two-dimensional areas of intracellular parasites, and infected and uninfected RBC were determined from microscope images (DIC channel) at up to 30 min after treatment with PDIP-1 (Fig. 5a). A higher PDIP-1 treatment concentration was used (38 µM) to ensure complete DV degradation. In untreated samples, the mean parasite area (± SD) was 11.9 ± 0.93 µm^2^, but after 1 min of treatment the area was increased by approximately 16% (13.8 ± 1.8 µm^2^) and by 30% after 10 min (15.4 ± 1.3 µm^2^). The parasite areas measured at the later time points (up to 30 min) were comparable to the 10 min value, and they did not exhibit any additional morphological changes (Fig. 5b). In contrast to the parasites, the areas and morphologies of the infected RBC (containing the enlarged post-treatment parasites) were not changed following the treatment (Fig. 5c). Similarly, the uninfected RBCs (present in the same treated samples) were not affected (Fig. 5d). Thus, treatment with PDIP-1 caused a rapid and sustained enlargement or swelling of parasites residing inside RBCs, but the RBCs themselves were unchanged. The onset of the parasite swelling appeared to correspond with DV destruction (see Video S3). To explore this further, experiments were conducted using PMII-GFP expressing parasites (trophozoites) treated with PDIP-1 (20 µM), in which the time of the DV loss was noted, and the area of the same parasite was compared one minute before and at one and fifteen minutes after the event. Before DV destruction the mean parasite area was 16.9 ± 4.0 µm^2^, whereas at one minute after this had increased by approximately 40% (24.0 ± 6.2 µm^2^) and by 66% after fifteen minutes (28.0 ± 8.0 µm^2^) (Fig. 5e). Therefore, parasite swelling occurred almost immediately after the DV was destroyed by the peptide.

**Fig. 6:**
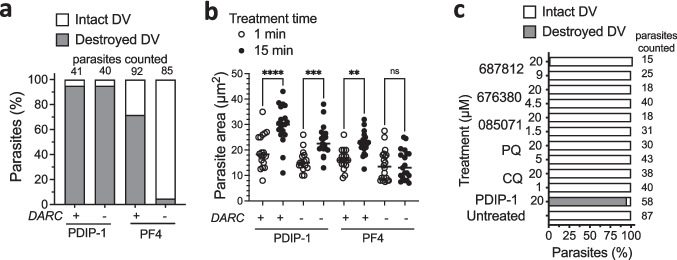
Digestive vacuole (DV) destruction and parasite size in DARC-positive and DARC-negative RBC following treatment with PDIP or PF4, and comparison to the lack of DV-destroying activity of other antiplasmodial compounds. **a**. Proportions of *P. falciparum* PMII-GFP infected RBC with unaffected (intact) and destroyed DV, cultured in either DARC-expressing (DARC +) or DARC-negative (DARC-) RBC after ten minutes treatment with 20 µM PDIP-1 or 10 µM PF4. Values above indicate total numbers of cells analyzed in each condition. **b**. The areas of intracellular *P. falciparum* 3D7 parasites grown in DARC + or DARC- RBC determined one and fifteen minutes after treatment with 20 µM PDIP-1 or 10 µM PF4. Shown are the measures for individual cells and means (horizontal lines) from three independent experiments. Comparisons were determined using ANOVA with Sidak’s corrections for multiple testing. ns, *p* > 0.05; **, *p* < 0.01; *** *p* < 0.001; ****, *p* < 0.0001. **c**. Proportions of *P. falciparum* PMII-GFP infected RBC with intact and destroyed DV, either untreated or 10 min after treatment with PDIP-1, chloroquine (CQ), primaquine (PQ) and three MMV compounds (with previously reported DV activity [42]) at the indicated concentrations. Values on the righthand side indicate total numbers of parasites analyzed in each condition from at least three independent experiments.

We have previously shown that PF4 is less active against parasites cultured in DARC-negative RBC compared to DARC-expressing RBC [24, 26]. PDIP analogs do not contain the chemokine region required by PF4 for interacting with DARC, therefore, we expected the DV-destructive and parasite swelling actions of the peptides (which do not contain the chemokine sequence present on PF4) to be insensitive to DARC expression. To investigate, *P. falciparum* PMII-GFP parasites were cultured in DARC-positive and DARC-negative RBCs, and the frequency of DV loss and the parasite size (two-dimensional area) were measured in samples treated with either PDIP-1 (20 µM) or PF4 (10 µM), as described above. Treatment with PDIP-1 resulted in DV destruction in almost all parasites for both cell types (approximately 95%), indicating no dependence on DARC. In contrast, treatment with PF4 caused DV destruction in approximately 75% of DARC-positive parasites but only 5% of DARC-negative parasites (Fig. 6a). Comparable effects were observed for parasite swelling, where significant increases in the parasite size were observed in both DARC-positive and DARC-negative cells treated with PDIP-1, and in DARC-positive but not DARC-negative cells treated with PF4 (Fig. 6b). These findings indicate that while PF4 and PDIP-1 have the same DV destruction and parasite swelling activities, the action of the chemokine-containing protein is DARC-dependent, whereas the uptake and action of the peptide is independent of DARC.

Using the same PMII-GFP parasites and real time microscopy method as described above, we also tested more than 100 compounds for their ability to induce DV destruction. This library included primaquine, chloroquine and three Medicines for Malaria Venture (MMV) compounds that have reported DV-directed killing mechanisms [42], several known antimalarial drugs and other antimicrobials, and additional MMV compounds with uncharacterized antiplasmodial activity. Under the same treatment conditions and time course (10 min) used in the above experiments, neither primaquine, chloroquine, or the three MMV compounds with previously reported DV activity, tested at concentrations sufficient to kill 100% of the cultured parasites (IC_100_) or at supra-IC_100_ levels (20 µM) were able to cause destruction of the DV comparable to PDIP-1 (Fig. 6c). Likewise, none of the remaining compounds tested (all at 10 µM) exhibited any DV-destructive activity (Table S2), thereby underscoring the unique action of PDIPs and distinguishing them mechanistically from other antimalarial agents.

## Discussion

PF4-derived peptide analogs were previously identified as effective and potent blood stage antiplasmodial agents, that were distinguished from current antimalarial drugs due to their origin from an innate human defense protein, their structural homology to AMPs, membrane-active properties, specificity for parasite infected RBC, and cytotoxic mechanism involving destruction of the parasite DV [22–24, 26, 27]. Here we employed newly developed real-time protocols that used live *P. falciparum*-infected human RBC to investigate the infected cell selective uptake and DV-destructive actions of two near identical and prototypical PF4 peptide analogs, PDIP-1 and PDIP-3 (from ref [27]), and provide previously lacking mechanistic detail and expand our understanding of the molecular requirements for their function. Studies on RBCs treated with a PDIP-3 fluorophore conjugate (PDIP-A488) using real-time microscopy in conjunction with flow cytometry demonstrated the peptide’s selectivity for infected cells, defined the kinetics of its entry and accumulation, and indicated that the composition of the infected host cell membrane (specifically levels of PS), but not a range of parasite membrane transporters, were important for its function. Additional experiments showed the macrocyclic structure of the PDIP scaffold is necessary and sufficient for its internalization and cytotoxic actions. A live cell video microscopy protocol was developed to visualize and temporally document, in live parasite-infected cells treated with the PDIPs or PF4, the rapid destruction of the DV and a concomitant swelling (but not lysis) of the parasite. The integrity of the host infected cell was not compromised following these events, but phospholipids in the plasma membrane were reorganized to increase levels of PS in the outer leaflet. In addition, the DV destructive event could not be detected in a library of > 100 compounds, collectively expanding our understanding of the remarkable and unique antiplasmodial properties of PF4 and the PDIP molecular scaffold class.

Our observations of accumulation of a fluorophore-labelled PDIP derivative (PDIP-A488) within live *P. falciparum* parasites are consistent with previous immunolocalization studies (cells fixed and immunostained after treatment with PF4 or PF4-derived peptides) showing they are localized in the parasite cytosol [23, 24, 26]. To accumulate within *Plasmodium* that reside inside RBC, these molecules must translocate across the three membranes that separate the parasite cytosol from the external medium – the RBC plasma membrane, the parasite parasitophorous vacuole membrane, and the parasite plasma membrane – and through this action leave the membranes intact. Comparable membrane-translocating functions have been reported for many other AMPs that target prokaryotic cells and certain proteins that penetrate eukaryotic cells [43]. However, the ability of PF4-derived peptides to penetrate three separate membranes extends considerably the capabilities of these types of molecules. Given that PDIP-3 maintained an intact disulfide bond (and therefore macrocyclic structure) inside parasites, and that non-cleavable (permanently cyclized) PDIP-24 killed parasites with similar IC_50_ compared to PDIP-3, we can also infer that a cyclic structure is necessary and sufficient for the membrane translocation process. These results align with our earlier findings that the sequence and structure of PDIP analogs are critical for their cell selectivity and parasite cytotoxicity [27]. The ability to dispense with the disulfide bond, and instead stabilize the structure via backbone cyclization will be useful in our efforts to produce PDIP-drug conjugate molecules for the targeted delivery of antiplasmodial compounds into infected cells [36, 44]; as the cysteine residues can instead be employed as sites for conjugation to cargo molecules.

Why are *Plasmodium*-infected RBCs highly permissive to PF4-derived peptides? We showed that two major parasite ion transporters, PfATP4 and V-type H^+^-ATPase, which regulate sodium and proton gradients, and the parasite NPP nutrient transport system were not required for PDIP-A488 uptake into parasite-infected cells. Instead, we found that the level of PS exposed on the surface of RBCs was important. PDIP analogs have a higher binding affinity for membranes containing PS [26, 27] and here we confirmed that infected cells have more exposed PS than uninfected cells, in agreement with earlier studies [29, 30]. When exposed PS was blocked, peptide (PDIP-A488) uptake was partly inhibited, and when PS exposure was increased following treatment with apoptosis-inducing drugs (in both uninfected or infected cells) peptide uptake was also increased. Additionally, we found that PS exposure on infected cells (but not uninfected cells) was further increased by treatment with PDIP-3, raising the possibility that PS-dependent peptide uptake can be amplified via a positive feedback mechanism. However, additional non-PS factors also likely contribute to internalization, as peptide (PDIP-A488) uptake could only be partially inhibited by blocking exposed PS, and it was also lower in PS-enriched uninfected cells than infected cells. Additional factors that mediate the uptake of PDIPs could include other membrane lipids known to be enriched in infected RBC that they can bind [31, 45], as well as other parasite-expressed membrane transporters that we did not investigate [46].

Live cell microscopy studies were used to document how PF4 and PDIPs rapidly destroy the DV of *P. falciparum* parasites inside host RBCs. DV destruction, which is considered the definitive cytotoxic event induced by these molecules [23, 24, 26], was completed following only 5–10 min treatment with the PDIP analogs. To our knowledge, this is the only group of antiplasmodials for which the real-time effects and activity kinetics have been described and may be the fastest known killers of *Plasmodium*. A third event, observed almost immediately following PDIP-induced DV destruction, was a 20–50% increase in the size of the parasite (residing inside the intact host cell). We presume this to be a consequence of the parasite cytosol mixing with the contents of the DV, which could affect the cytosolic osmolarity or alter the ionic composition of the parasite (e.g. reduced pH [26]) and disturb other mechanisms that regulate parasite volume. A similar parasite swelling effect has been seen in parasites exposed to PfATP4 inhibitors, because of sodium entering the parasite and increasing the osmotic pressure [47]. However, these observations were made in parasites removed from the host cell and the effect took longer to occur (10–20 min after inhibitor treatment), suggesting that different mechanisms are involved. We also showed that PF4 induced a similarly rapid DV destructive and cell swelling activity to PDIP-1, indicating its uptake kinetics and cytotoxic mechanism are similar. A side-by-side analysis of DV destruction and parasite swelling in DARC-expressing versus DARC-negative RBCs further demonstrated (and confirmed [24, 26]) that DARC is essential for PF4’s activity, while PDIPs operate independently of DARC.

Another significant finding of this study was that the internalization and DV-destructive actions of the PDIPs and PF4 did not visibly damage the integrity of the host cell. Apart from the parasite swelling and increased exposure of PS on the surface of treated RBCs, both parasite and host cell plasma membranes remained intact. In contrast, other AMP-like and membrane-active peptides with antiplasmodial activity, such as NK-2 and the dermaseptins [48, 49], act by lysing the entire infected cell. This difference emphasizes PF4 as a highly adapted and refined host defense molecule that can act on an intracellular pathogen whilst minimizing host cell damage and exposing pathogen components. Notably, PF4-containing but otherwise intact *Plasmodium* infected cells are observed in the circulation of patients with malaria (because of the targeted actions of platelets, [22]). If comparable enhancement of PS-exposure occurs in platelet- and PF4-exposed cells in vivo, this may also enhance their phagocytic recognition and clearance [50], and thereby link two important arms of innate immunity, platelets and phagocytes, in the host protection against malarial infection.

In conclusion, this study demonstrates in real time the selective and rapid internalization and DV destructive action of PDIPs, an AMP-like class of peptide derived from human PF4, in live *Plasmodium*-infected RBCs. The ability of PDIPs to traverse multiple membranes without compromising host cell integrity differentiates them from other AMPs and underscores their therapeutic potential. These findings not only position PDIPs as a promising new class of antiplasmodials but also suggest value for including them as part of a broader strategy to enhance the efficacy of existing therapies, leveraging human defense mechanisms to combat malaria more effectively.

## Supplementary Information

Below is the link to the electronic supplementary material.

Supplementary file1 (PDF 1270 KB)

Supplementary file2 (AVI 536 KB)

Supplementary file3 (AVI 495 KB)

Supplementary file4 (M4V 89 KB)

## Data Availability

The datasets generated and analyzed during the current study can be obtained through request to the corresponding authors.
