# Supplementary material for: Insights into platelet factor 4-derived peptide macrocycles; the mechanistic basis of their rapid and selective antiplasmodial actions
Source: Cell Mol Life Sci. 2025 Jun 9;82(1):228. doi: 10.1007/s00018-025-05757-y (PMC12149375; doi:10.1007/s00018-025-05757-y)
Supplement: Supplementary file 1 — Supplementary file1 (PDF 1270 KB) [file 18_2025_5757_MOESM1_ESM.pdf]

Supplementary data

Tables S1 and S2
Figures S1-S6
Supplementary references

Table S1. PDIP-A488 peptide internalization and accumulation kinetics in *P. falciparum* 3D7 infected RBC.

PDIP-A488 concentration (μM)	Time for 100% PDIP-A488 internalization (min ± SD)[#]	Maximal PDIP-A488 accumulation (A488 MFI ± SD)[##]
2.5	40.5 ± 0.6	2060 ± 410
5	7.3 ± 0.3	3260 ± 190
10	5.4 ± 0.8	4810 ± 390
20	5.3 ± 0.6	6830 ± 230

[#] Minimum time required for 100% of *P. falciparum*-infected cells to internalize PDIP-A488 peptide, calculated using the data in Figure 1C and non-linear regression.

[##] Maximal possible PDIP-A488 peptide accumulation levels in *P. falciparum*-infected cells, calculated using the data in Figure 1D and non-linear regression. MFI, mean fluorescence intensity.

Table S2. List of the compounds tested for digestive vacuole destructive activity.

Compound ID #	3D7 EC50 (µM) ##
MMV010764	1.4
MMV000907	1.1
MMV084603	1.2
MMV1028806	>2
MMV676350	0.8
MMV026020	0.5
MMV006372	0.8
MMV011903	0.4
MMV020591	1
MMV020623	0.9
MMV020512	0.5
MMV020982	0.9
MMV020120	0.6
MMV676605	>2
MMV007638	0.7
MMV021057	0.1
MMV020136	1.1
MMV020710	0.2
MMV020517	0.9
MMV019721	0.5
MMV020537	1
MMV019838	1.1
MMV020520	1.1
MMV019234	0.3
MMV016136	0.6
MMV676442	0.8
MMV020152	0.8
MMV024397	1.3
MMV019807	1.7
MMV560185	>2
MMV019189	1.1
MMV020321	1.4
MMV019087	0.2
MMV676528	1.6
MMV020320	0.7
MMV085210	>2
MMV026313	0.2
MMV392832	>2
MMV084864	0.5

Compound ID #	3D7 EC50 (µM) ##
MMV006239	0.5
MMV000858	1.2
MMV006741	0.6
MMV019742	1
MMV009054	1.9
MMV006901	0.6
MMV020391	0.9
MMV676380	0.4
MMV008439	>2
MMV020388	0.5
MMV022236	0.2
MMV1030799	>2
MMV021375	1.4
MMV1029203	>2
MMV062221	>2
MMV1088520	>2
MMV023370	0.4
MMV1019989	>2
MMV1037162	>2
MMV011229	0.4
MMV393144	>2
MMV007920	1.5
MMV019993	0.1
MMV687794	0.3
MMV023183	0.6
MMV020165	0.3
MMV667494	0.007
MMV028694	1.1
MMV010545	0.5
MMV023227	1.9
MMV020289	0.9
MMV634140	>2
MMV030734	0.4
MMV676358	1.5
MMV407834	>2
MMV019551	0.2
MMV016838	0.1
MMV676270	NA

MMV reference compound #
Bedaquiline
Pentamidine
Clofazimine
alpha-Difluoromethylornithine
Suramin
Sitamaquine
Benznidazole
Doxycycline
Posaconazole
Nitazoxanide
Ethambutol
Buparvaquone
Mebendazole
Levofloxacin (-)-ofloxacin
Rifampicin
Amphotericin B
Nifurtimox
Diethylcarbamazine
Amikacin
Auranofin
Miltefosine

P. falciparum PMII-GFP infected red blood cells were treated with 10 µM of each compound for 10 minutes and examined for signs of digestive vacuole destruction according to the methods described in the main text. No DV-disruptive activity was observed for any of the compounds listed.

MMV compound concentrations of required to inhibit 50% growth of P. falciparum 3D7 blood stage parasites (EC50) (https://chembl.gitbook.io/chembl-ntd)

Figure S1

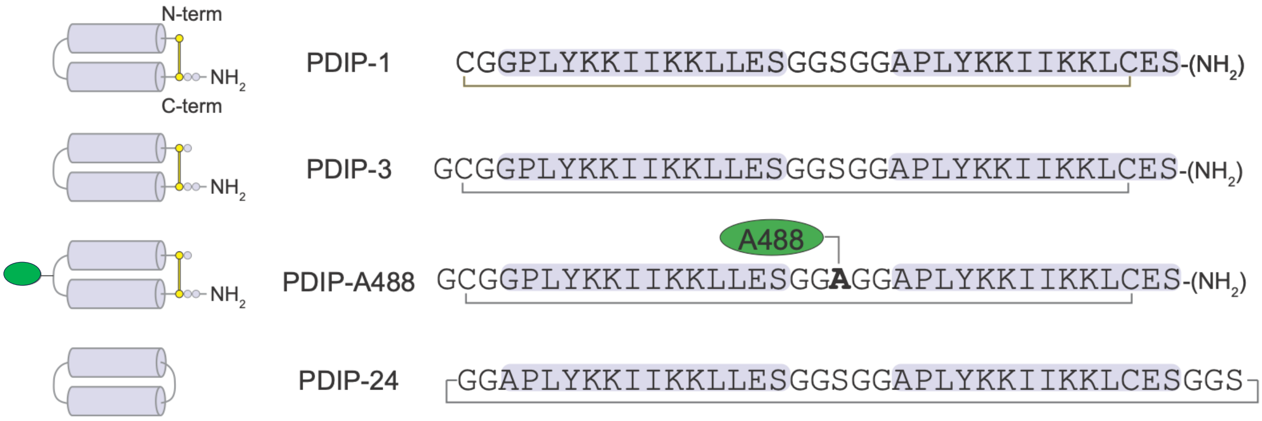

Figure S1. PF4-derived internalization peptide (PDIP) analogs used in this study.
Cartoon representation of the structures and sequences of the PDIP analogs used in these studies. PDIP analog 1 (PDIP-1) was previously named cPF4PD and described in [1]. PDIP analogs 3 and 24 (PDIP-3 and PDIP-24, respectively) and Alexa Fluor 488-labelled PDIP (PDIP-A488) were described in [2]. The bold A residue indicates an azidoalanine amino acid used to attach A488.

Figure S2

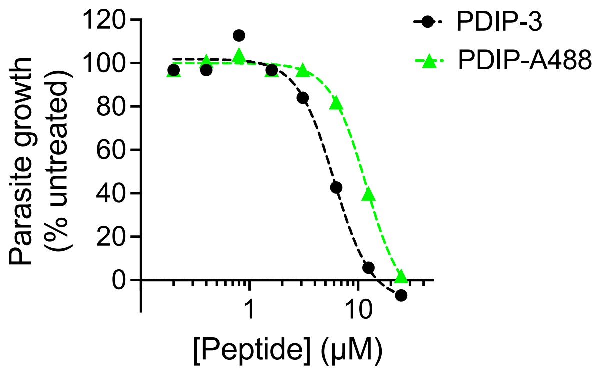

Figure S2. Dose response curve showing *in vitro* growth inhibition of *Plasmodium falciparum* 3D7 parasites treated with PDIP-3 or PDIP-A488.
Data points are single replicates from a representative experiment. Curves were fitted using GraphPad Prism v 10.0.2 [inhibitor] versus response with four parameters and constraining the top of the curve to 100%.

Figure S3

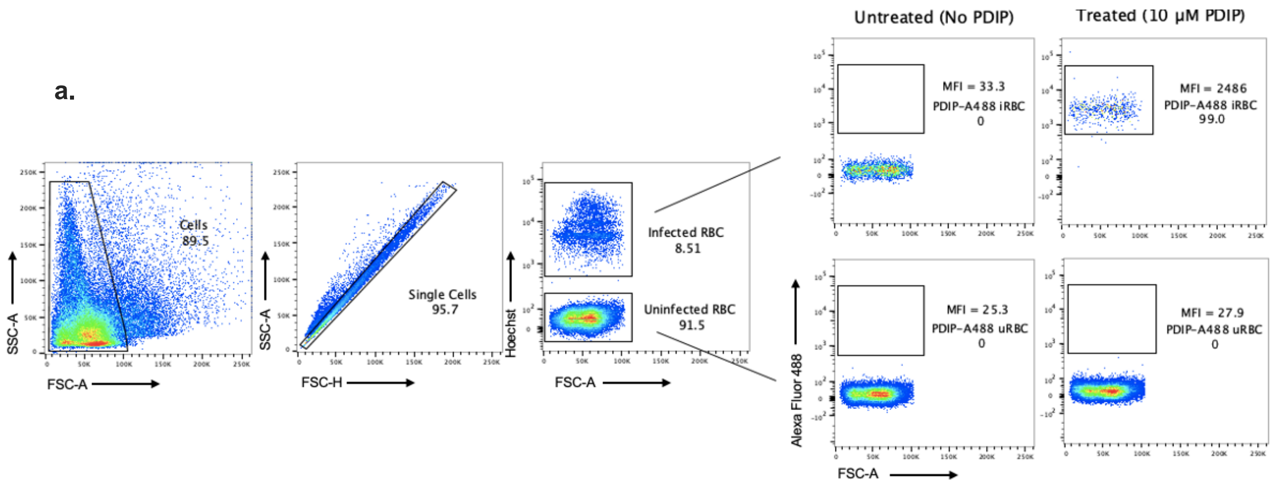

Figure S3. Flow cytometry gating strategy for PDIP-A488 peptide internalization analysis.
P. falciparum 3D7 infected and uninfected human RBC were washed, stained with Hoechst 33342 for 20 minutes at 37°C, treated with PDIP-A488 peptide at indicated concentrations and analysed using flow cytometry. The depicted gating strategy was used to identify single cells, infected and uninfected cells, and peptide positive and negative populations in each cell type. The A488 MFI was determined separately in the infected and uninfected cells.

Figure S4

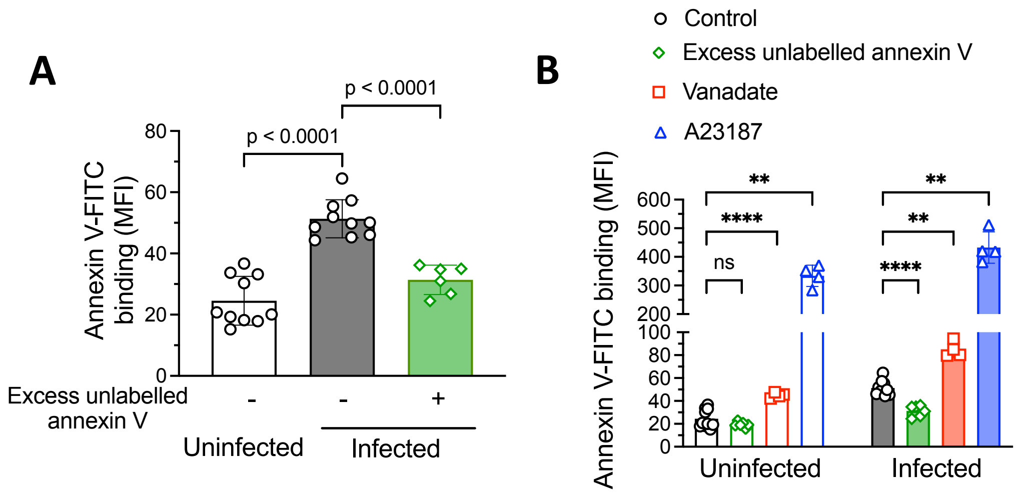

Figure S4. Annexin V-FITC binding in *P. falciparum* infected and uninfected RBC and following treatment with sodium orthovanadate or A23187.

A. Membrane-bound annexin V-FITC levels (mean fluorescence intensity, MFI) in uninfected RBC, and *P. falciparum* 3D7 infected red blood cells with and without 10-fold molar excess unlabeled annexin V (40 µg/mL). Annexin-FITC levels were approximately two times higher on infected cells compared to uninfected cells and excess annexin V significantly reduced annexin-FITC binding to infected cells, demonstrating the binding specificity. **B.** Annexin V-FITC binding levels (MFI) on uninfected and infected RBC with and without 10-fold molar excess of unlabeled annexin V (same data shown in A), 500 µM sodium orthovanadate (vanadate) or 2 µM A23187 compared to untreated (control) cells. Vanadate inhibits ATPase-dependent membrane phospholipid translocases and flippases, whose activities deplete PS in the outer membrane leaflet of RBC, and A23187, a calcium ionophore, activates membrane scramblases and causes enrichment of PS outer membrane leaflet of RBC [3]. Compared to control cells, treatment with unlabeled annexin V significantly reduced annexin V-FITC binding on infected RBC (by approximately 2 times lower), while binding on uninfected cells was unchanged. Treatment with vanadate significantly increased annexin V binding on both infected and uninfected cells (approximately 2 times higher MFI compared to respective controls), and A23187 increased annexin V binding 14 times on uninfected cells and 8 times higher MFI for infected cells. Shown are replicate measures, means (bars) and SD (error bars) from 2-4 independent experiments. Comparisons were determined using using one-way ANOVA with Holm-Sidak's correction for multiple comparisons; ns, $p > 0.05$; ** $p < 0.01$; **** $p < 0.0001$.

Figure S5

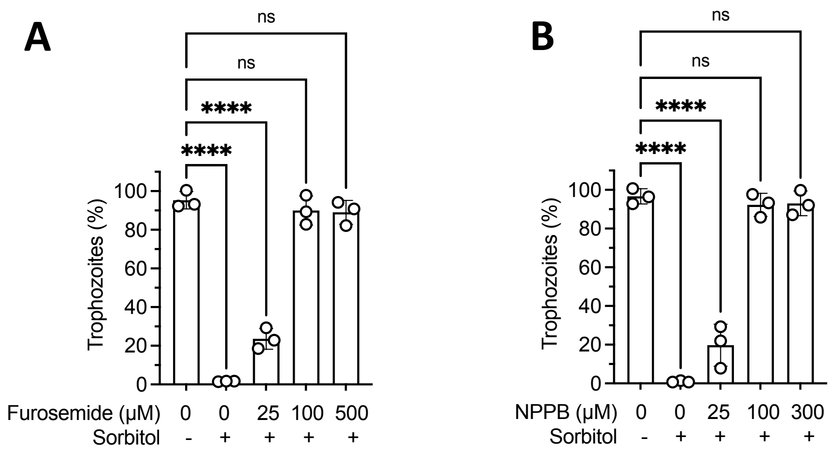

Figure S5. New permeation pathway (NPP) inhibition by furosemide and NPPB.
Unsynchronized cultures of *P. falciparum* 3D7 infected RBC were treated with different concentrations of furosemide (**A**) or 5-nitro-2-(3-phenylpropylamino) benzoic acid (NPPB, **B**) for 10 min, and then washed and incubated in 5% sorbitol or medium without sorbitol for 10 min and the proportions of trophozoite-stage infected cells evaluated in Giemsa-stained blood smears under light microscopy. NPP-sufficient parasites are susceptible to sorbitol induced lysis, while parasites in which NPP is inactivated are resistant to sorbitol lysis. Shown are measures normalized to the untreated control samples from three replicates and means (bars) in a representative experiment. Comparisons to trophozoites in untreated samples without sorbitol using one-way ANOVA with Dunnett's test for multiple comparisons. ns, $p > 0.05$; ****, $p < 0.0001$. The two highest concentrations of each compound prevented sorbitol-induced lysis.

Figure S6

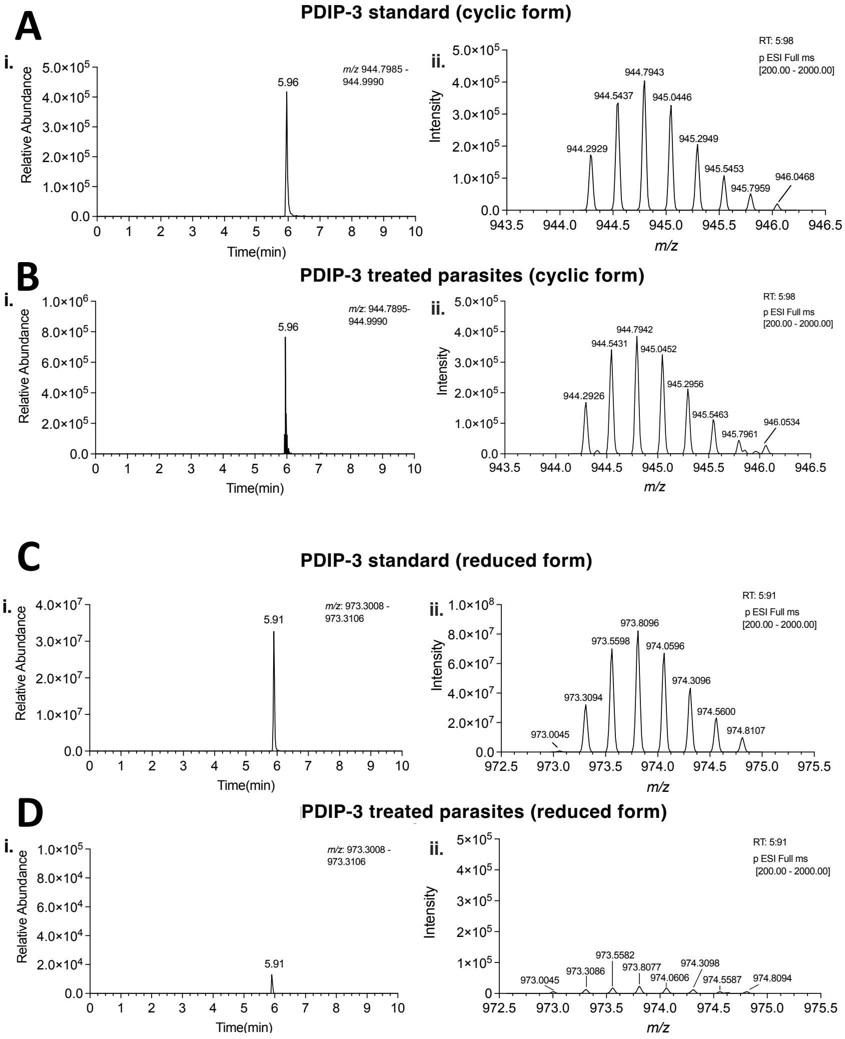

Figure S6. HPLC-MS analysis of cyclic (oxidized) and linear (reduced) PDIP-3.
Representative ion chromatograms (**i**. HPLC elution peak; **ii**. MS ion isotopic spectra) for purified forms of cyclic and reduced (linear) PDIP analog 3 (PDIP-3) standards (**A** and **C**, respectively), and corresponding detection of each peptide form (**B** and **D**, respectively) in *P. falciparum* infected RBC treated with 10 µM PDIP-3 for 5 minutes and then washed 3 times with 100x volume culture medium prior to protein extraction. Cyclic PDIP-3 was quantified by integration of the signal area for the most abundant peak (m/z of 944.79). To detect and quantify reduced PDIP-3, an iodoacetamide-tagged version was prepared as a standard, and the integrated signal area for the most abundant peak (m/z of 973.81) was determined in iodoacetamide-treated parasite extracts.

Supplementary References

1. Lawrence, N., et al., *Defense Peptides Engineered from Human Platelet Factor 4 Kill Plasmodium by Selective Membrane Disruption.* Cell Chem Biol, 2018. **25**(9): p. 1140-1150.e5. 10.1016/j.chembiol.2018.06.009
2. Lawrence, N., et al., *Enhancing the Intrinsic Antiplasmodial Activity and Improving the Stability and Selectivity of a Tunable Peptide Scaffold Derived from Human Platelet Factor 4.* ACS Infect Dis, 2024. **10**(8): p. 2899-2912. 10.1021/acsinfecdis.4c00276
3. Fraser, M., et al., *Breakdown in membrane asymmetry regulation leads to monocyte recognition of P. falciparum-infected red blood cells.* PLoS Pathog, 2021. **17**(2): p. e1009259. 10.1371/journal.ppat.1009259
